# Unnecessary Operations on Women

**Published:** 1906-12

**Authors:** 


**A Monthly Review of Medicine and Surgery.**

*EDITOR:*

WILLIAM WARREN POTTER, M. D.

All communications, whether of a literary or business nature, books for review and exchanges, should be addressed to the editor 284 FRANKLIN ST., BUFFALO, N. Y.

VOL. LXII. DECEMBER, 1906. NO. 5

### "Unnecessary Operations on Women."

UNDER this title elsewhere in this issue will be found an interesting correspondence between Dr. Arthur Dean Bevan, of Chicago, and Dr. Lewis S. McMurtry, of Louisville. Dr. Bevan's letter was published, as will be observed, in the October number of "Surgery, Gynecology, and Obstetrics," and Dr. McMurtry's answer appeared in the November edition of the same magazine. We republish both letters in this Journal because of the gravity of the charges made by Dr. Bevan against a large and respectable class of the profession, and of the injury likely to result; and because, further, of the complete refutation of the charges by Dr. McMurtry.

The propriety of Dr. Bevan's action in writing such a letter may well be questioned. Is it not quite enough that the profession should be assailed, villified, and held up to scorn from without? Or, must it also be subjected to a similar attack within its own ranks? If Dr. Bevan is aware of the evils which he describes, let him speak specifically, and not condemn a large group of trained gynecologists who are the peers of any other group of specialists in the land, because of the blunders of a few neophytes who may have taken inspiration from a few weeks' attendance at a post-graduate school, and could not be entitled thereby to call themselves specialists in gynecology.

Dr. Bevan is a surgeon of distinction, a teacher in a great medical institution, and chairman of the Council on Medical Education of the American Medical Association. Whatever he may say, therefore, naturally carries great weight, hence all the more caution is required in speaking to the professional world through the columns of a great medical magazine. We are not informed as to his history but he writes like one who has never had experience in general medicine, but began his surgical career as soon as his undergraduate work was ended. There are many such and they lack the broad training afforded by years of general practice. They are intolerant as a general thing of the views of others, and assume an imperious air in the presence of those not of their inclining.

How completely the charges have been refuted may be determined by a careful reading of Dr. McMurtry's letter. It is a logical, courteous, conclusive answer to the indictment and should make the assailant quite ashamed of his inconsiderate utterances. While, as we have stated, the answer is polite in diction it, nevertheless, betrays indignation, as everyone who knows Dr. McMurtry can observe without even reading between the lines. The profession should be profoundly thankful that the accomplished ex-president of the American Medical Association has taken up the challenge and met every detail at issue in such conclusive fashion. We hope the correspondence will be read with care by general and gynecological surgeons as well as by those who practise general medicine.

